# Transplantation of Stem Cells from Human Exfoliated Deciduous Teeth Decreases Cognitive Impairment from Chronic Cerebral Ischemia by Reducing Neuronal Apoptosis in Rats

**DOI:** 10.1155/2020/6393075

**Published:** 2020-03-06

**Authors:** Shu Zhu, Dongyu Min, Jianhong Zeng, Yetao Ju, Yao Liu, Xu Chen

**Affiliations:** ^1^Department of Pediatric Dentistry, School of Stomatology, China Medical University, Shenyang 110002, China; ^2^Liaoning Provincial Key Laboratory of Oral Diseases, Shenyang 110002, China; ^3^The Affiliated Hospital of Liaoning University of Traditional Chinese Medicine, Shenyang 110032, China

## Abstract

Stem cells from human exfoliated deciduous teeth (SHED) are a unique postnatal stem cell population with high self-renewal ability that originates from the cranial neural crest. Since SHED are homologous to the central nervous system, they possess superior capacity to differentiate into neural cells. However, whether and how SHED ameliorate degenerative central nervous disease are unclear. Chronic cerebral ischemia (CCI) is a kind of neurological disease caused by long-term cerebral circulation insufficiency and is characterized by progressive cognitive and behavioral deterioration. In this study, we showed that either systemic transplantation of SHED or SHED infusion into the hippocampus ameliorated cognitive impairment of CCI rats in four weeks after SHED treatment by rescuing the number of neurons in the hippocampus area. Mechanistically, SHED transplantation decreased the apoptosis of neuronal cells in the hippocampus area of CCI rats through downregulation of cleaved caspase-3. In summary, SHED transplantation protected the neuronal function and reduced neuronal apoptosis, resulting in amelioration of cognitive impairment from CCI. Our findings suggest that SHED are a promising stem cell source for cell therapy of neurological diseases in the clinic.

## 1. Introduction

Chronic cerebral ischemia (CCI) is considered both a neurological and cerebrovascular disease and is characterized by progressive cognitive and behavioral deterioration caused by long-term cerebral blood perfusion insufficiency. It is closely related to many cerebrovascular diseases, including cerebral arteriosclerosis, cerebral infarction, vascular dementia (VaD), and Alzheimer's disease. CCI is a major cause of disability and constitutes a large healthcare burden. Multiple factors are implicated in CCI, and the mechanisms involved are not fully understood. It may be associated with oxidative stress, apoptosis, inflammatory response, synaptic dysfunction, and energy metabolism disorders. Studies have shown that hippocampal neurons in CCI display a loose arrangement with reduced numbers and irregular morphology and higher levels of apoptosis have been observed in the hippocampus of CCI rats [1, 2]. The current clinical treatment for CCI is still drug administration. Medication could suppress brain NF-*κ*B activity, reduce neuronal apoptosis and autophagy, and increase the Bcl-2/Bax ratio to induce neuroprotection in a CCI model [3–5]. However, even the most potent neuroprotective drugs have been shown to be ineffective for reversing neuronal damage in brain tissue. Therefore, it is important to identify new effective strategies to treat CCI.

Mesenchymal stem cells (MSCs) possess self-renewal and multipotential differentiation abilities. Systemic MSC transplantation (MSCT) has been successfully used to treat a variety of human diseases, such as systemic lupus erythematosus, myocardial infarction, and inflammatory bowel disease [6, 7]. Recently, studies have reported that MSCT promoted neurorecovery and ameliorated ischemic brain injuries [8, 9]. MSCT restored memory through promoting endogenous neurogenesis and synaptic remodeling in AD mice and improved early cognitive functions and daily living activities in VaD patients [10, 11]. MSCT has been identified as a new strategy for treating cerebrovascular diseases. Multiple therapeutic mechanisms may underlie the effects of MSCT-based therapies, including direct differentiating into functional neurons, paracrine effects, and interplay between MSCs and immune cells. However, the detailed mechanisms are not fully understood.

Stem cells from human exfoliated deciduous teeth (SHED) are a unique postnatal stem cell population with high self-renewal ability that originates from the cranial neural crest. Because they are homologous to the central nervous system, SHED express both mesenchymal and neuroectodermal markers. Moreover, obtaining SHED from the pulp of deciduous teeth is atraumatic; further, SHED are associated with minimal ethical concerns regarding their extraction and use [12]. SHED exhibit a higher proliferative activity and neural differentiation ability [13] and may be capable of differentiating into neurons, dopaminergic neurons, sensory neurons, and retinal photoreceptor-like cells *in vivo* [14–16]. A recent study reported that SHED were capable of regenerating functional dental pulp with blood vessels and nerves in a large animal model. Moreover, SHED could lead to regeneration of 3D whole dental pulp tissue containing an odontoblast layer, connective tissue, neuron, and blood vessels, similar to normal dental pulp [16]. It has been confirmed that SHED transplantation can be an effective treatment for nervous system diseases. SHED grafts could promote functional recovery after spinal cord injury, and they could differentiate into functional neurons and oligodendrocytes [17–19]. SHED could also reduce neuroinflammation by shifting microglia polarization through paracrine effects, thus improving motor functional recovery and reducing cortical lesion in rats with traumatic brain injury [20]. However, whether SHED transplantation has treatment effects on CCI and the underlying mechanisms are still unclear.

In this study, we used a rat model with two-vessel occlusion, a classical CCI model, to investigate the treatment effects of SHED transplantation, in which memory and neuronal functions of a CCI model were evaluated, and we further explored the underlying mechanisms. Our data showed that SHED transplantation decreased cognitive impairment of CCI rats and protected the cell functions of neurons by reducing cell apoptosis.

## 2. Materials and Methods

### 2.1. Animals and Ethics Statement

Male Wistar rats weighing 200–250 g were purchased from the Liaoning Changsheng Biotechnology Corporation (Benxi, China). All animals were acclimated to the environment under temperature-controlled conditions and a 12 h light/dark cycle for 1 week with free access to food and water. All study protocols were approved by the Ethics Committee of the School of Stomatology at China Medical University, Shenyang, China (No. 2018099).

### 2.2. CCI Model

In this study, a CCI model was induced by following the previously reported protocol [21, 22]. In brief, after rats were anesthetized with 0.1% (*w*/*v*) sodium pentobarbital (40 mg/kg intraperitoneally; Cristália, Itapira, Brazil), both carotid arteries were gently separated from the vagus nerve, and permanent artery occlusion was implemented with a 5-0 silk thread. The sham-operated rats were treated in a similar way, but without occlusion through double ligation. A successful CCI model was defined as marked decreases of cerebral blood flow velocity at both end diastole and systole, which was confirmed by a small animal ultrasound (Supplementary [Supplementary-material supplementary-material-1]). After 4 weeks, rats were subjected to the Morris water maze (MWM) test to evaluate the cognitive deficit.

### 2.3. Isolation and Characterization of SHED

The donors of SHED, or their legal guardians in the case of minors, provided signed informed consent. Deciduous teeth were extracted and transported to the laboratory in Minimum Essential Medium Alpha (*α*-MEM; Gibco, Invitrogen, Carlsbad, CA, USA) solution containing 300 U/mL penicillin and 300 *μ*g/mL streptomycin (Hyclone, Logan, UT, USA) maintained at 4°C. Under aseptic conditions, the pulp chamber and root canal were exposed by cutting around the cement-enamel junction. Then, the dental pulp was isolated using a barbed broach. The dental pulp was minced and cultured in a 5% CO_2_ atmosphere at 37°C in a 10 cm culture disk containing *α*-MEM, 15% fetal bovine serum (FBS; MRC, Jiangsu, China), 100 U/mL penicillin-streptomycin (Hyclone), and 0.1 mmol/L L-ascorbic acid (Sigma-Aldrich, St. Louis, MO, USA). Half of the medium was replaced after 7 days, and all the medium was replaced every 3 days thereafter. Upon reaching 90% confluence, the cells were digested with 0.25% Trypsin-EDTA (Gibco). Cells at passages 3–5 were used for subsequent experiments.

SHED were analyzed for cluster of differentiation protein (CD) phenotypes. A total of 1 × 10^6^ cells/tube were blocked with 0.5% bovine serum albumin (BSA) and incubated with primary antibodies targeting CD73, CD90, CD105, CD34, and CD45 (1 : 100, Abcam, Cambridge, MA, USA) for 1 h in the dark on ice. Stained cells were neutralized with 0.5% BSA and fixed in 2% paraformaldehyde, then analyzed with a flow cytometer (Becton Dickinson, Franklin Lakes, NJ, USA). Next, SHED were differentiated to neuron-like cells. In brief, differentiation was induced in neural expansion medium Neurobasal A (Gibco) supplemented with 20 ng/mL basic fibroblast growth factor (bFGF, R&D) and 20 ng/mL human recombinant epidermal growth factor (EGF, R&D) supplemented with 2% human leukocyte antigen B27 (Gibco). Cells were grown in a humidified 5% CO_2_ atmosphere at 37°C. Medium was replaced daily. After 7 days, the stem cells were confirmed to positively express the neural cell marker *β*III-tubulin and neural stem cell-specific marker Nestin.

### 2.4. SHED Transplantation

CCI rats received SHED transplantation to the hippocampus (SHED-hippocampus groups) or through the tail vein (SHED-vein group). To identify the effect of SHED transplantation on hippocampal neuronal apoptosis, rats were randomly assigned to six groups: sham (healthy) group, ischemia group, SHED-hippocampus 2 × 10^4^ group, SHED-hippocampus 2 × 10^5^ group, SHED-hippocampus 2 × 10^6^ group, and SHED-vein 2 × 10^6^ group (*n* = 6 per group).

In the SHED-hippocampus groups, the skull was exposed through a midline skin incision and a burr hole was made using a small dental drill. Bilateral hippocampus areas were used as the injection site. The location of the incision relative to bregma was as follows: anteroposterior (AP): 3.2 mm, mediolateral (ML): ±2.0 mm, and dorsoventral (DV): 3.5 mm. After anesthesia, each rat was injected bilaterally with 10 *μ*L phosphate-buffered saline (PBS) or cell suspension at the lesion site with a microsyringe 24 h after inducing CCI. All rats were given transplants without immuno-suppression. The syringe remained in place for 5 min after the injection to allow the diffusion of the suspension into the surrounding tissue.

The SHED-vein group was treated with 2 × 10^6^ cells diluted in 150 *μ*L PBS, and SHED were injected by tail intravenous injection 24 h after inducing CCI. In addition, an equal volume of PBS was injected into rats in the sham group. Animals were sutured following standard surgical procedures and housed in individual cages until behavioral function was recovered. All rats were subjected to the MWM test again after 1 month.

### 2.5. MWM Test

CCI can be induced by ligating the bilateral common carotid arteries, causing neuronal sequelae such as cognitive impairment. The MWM test is a well-validated method for evaluating learning and memory in rats. It is a memory test that relies on the capacity of animals to rescue themselves by reaching a hidden goal platform in a pool of water [23]. Rats with neuronal damage generally display spatial memory impairments in the MWM test. The MWM test was conducted daily for 5 days in all rats. Each trial lasted until the rat being tested to locate the hidden escape platform within 1 min or less. If unsuccessful, the rat was guided by the tester to the hidden platform for 10 s. Escape latency was recorded as an assessment of spatial memory. After the last learning trial, on day 6, a probe trial was conducted to evaluate spatial memory. The platform was removed from the water, and each rat was allowed to swim freely for 60 s. To avoid a short-term memory in the MWM test, the behavior of each rat was recorded every 8 hours for three times, and the average value of these three results was recorded [24]. Rats were dried and returned to their cages after each trial. The mean values for escape latency and swimming speed in the daily trials were recorded, and the number of times rats crossed the platform region was recorded and defined as the spatial memory.

### 2.6. Neuropathological Analyses

After animals were anesthetized with pentobarbital (100 mg/kg intraperitoneally), they were sacrificed by transcardiac perfusion by injecting them with 0.9%, followed by 4% paraformaldehyde in 0.1 M PBS (pH 7.4). The brains of rats from each group were removed, embedded in paraffin, and sectioned into 4 *μ*m coronal sections on a sliding microtome. The sections were stained with Nissl, and images were captured using a digital camera. TUNEL staining was performed to evaluate hippocampal neuronal cell apoptosis. For TUNEL staining, sections were boiled by microwaving in a citrate buffer (10 mM, pH 6.4) for 5 min for antigen retrieval after deparaffinization and rehydration. Then, the sections were directly incubated with TUNEL mix from the *in situ* Cell Apoptosis Detection Kit V (POD) (Boster Biological Technology, Pleasanton, CA, USA) according to the manufacturer's protocol. Six sequential slices of the hippocampus were used with a 5 *μ*m interval between each two adjacent sections from each animal group to assess the number of pyramidal neurons in the CA1 region of the hippocampus.

### 2.7. Western Blotting

The hippocampus on one side was removed, homogenized in ice-cold lysis buffer, and centrifuged at 10,000 × g for 15 min at 4°C. Protein concentrations were analyzed using the bicinchoninic acid (BCA) Protein Assay Kit (Beyotime Biotechnology, Jiangsu, China). Samples with an equal amount of protein (50 *μ*g) were separated by SDS-PAGE and then transferred onto nitrocellulose membranes (Millipore, Billerica, MA, USA). The membranes were then blocked using 5% fat-free milk for 1 h and incubated overnight at 4°C with the following primary antibodies: rabbit anti-brain-derived neuronal factor (BDNF; 1 : 1000; Affinity Biologicals, Ancaster, Canada), rabbit anti-postsynaptic density protein 95 (PSD95; 1 : 1000; Proteintech, Rosemont, IL, USA), rabbit anti-synaptophysin (SYN; 1 : 1000; Proteintech), and rabbit anti-caspase-3 (1 : 1000; Cell Signaling Technology, Danvers, MA, USA). The membranes were washed with TBS-T, followed by incubation with horseradish peroxidase-conjugated goat anti-rabbit IgG (1 : 5000; Affinity Biologicals) for 2 h at room temperature. Immunoreactive bands were visualized by the enhanced chemiluminescence (ECL) kit (Pierce Biotechnology, Rockford, IL, USA) and exposed on an X-ray film. The immunoblot intensities were quantified using the Quantity One software (Bio-Rad Laboratories, Hercules, CA, USA).

**Figure 1:**
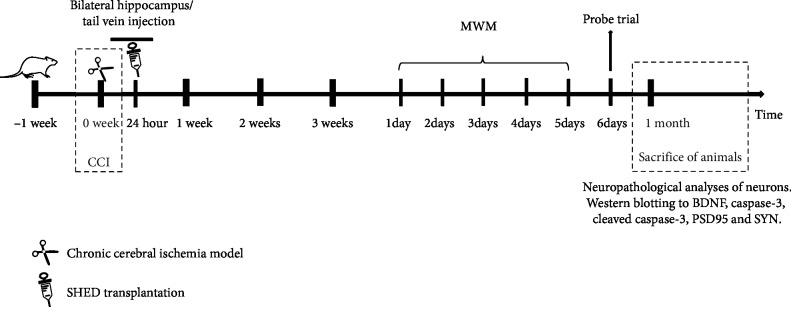
The schematic of the animal experiment. SHED transplantation was performed in CCI rats 24 hours after bilateral ligation of their carotid arteries. One of the two methods was used for the transplantation: rats were injected in either the hippocampus or the tail vein. During the final week of the experiment (fourth week), all animals were subjected to functional evaluation *via* the Morris water maze. One month after bilateral ligation of their carotid arteries, the animals were euthanized; samples were collected for morphological analysis to determine the number of surviving and apoptotic neurons in the CA1 region of the hippocampus. In addition, the expression levels of BDNF, caspase-3, cleaved caspase-3, PSD95, and SYN were analysed using western blot.

The process of experimental process of CCI rats was shown as the following schematic (Figure 1).

### 2.8. Statistical Analysis

Data were presented as the mean ± standard deviation (SD). All analyses were carried out using SPSS 17.0. Statistical comparisons were performed with one-way analysis of variance (ANOVA) followed by Tukey's post hoc test. Differences were considered statistically significant at *P* < 0.05.

## 3. Results

### 3.1. Characterization of SHED

**Figure 2:**
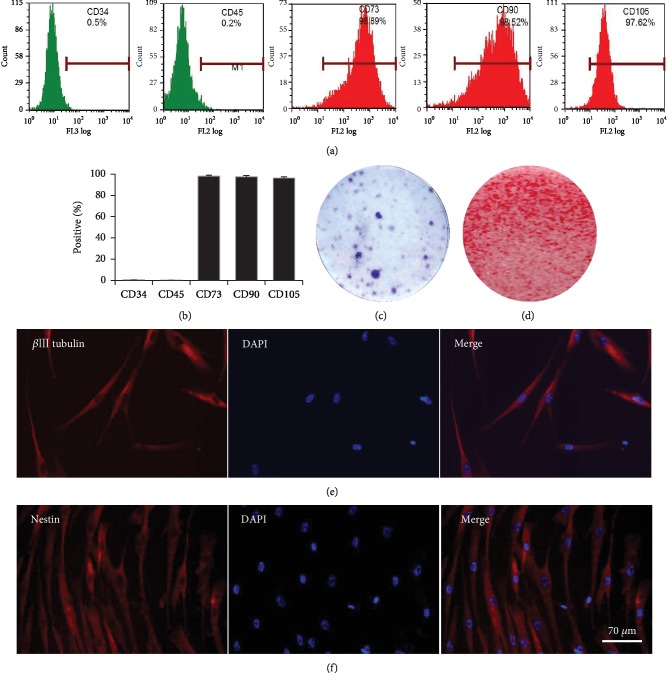
SHED express mesenchymal stem cell surface markers and can differentiate into osteogenic and neuron-like cells. (a, b) Expression of mesenchymal stem cell- (MSC-) specific surface markers in SHED. Positive expression was observed for CD73, CD90, and CD105, and negative expression was observed for CD34 and CD45. (c) Primary culture of SHED with CFU staining. (d) Alizarin red staining showed the mineralized nodule formation of SHED. (e, f) SHED differentiated into neuron-like cells, and expressed the neural cell marker *β*III-tubulin and the neural stem cell-specific marker Nestin. Scale bar: 70 *μ*m.

SHED were isolated and characterized *in vitro*. Flow cytometric analysis showed that SHED expressed MSC surface markers, such as CD73 (98.89%), CD90 (98.52%), and CD105 (97.62%) but did not express hematopoietic markers, such as CD34 (0.5%) and CD45 (0.2%) (Figures 2(a) and 2(b)). The cell colony formation of SHED was observed on the 14th day after primary culture (Figure 2(c)). Alizarin red staining showed that SHED were able to differentiate into the osteogenic lineage and formed mineralized nodules under osteogenic inductive conditions (Figure 2(d)). Immunofluorescence staining showed that SHED expressed neural markers, including *β*III-tubulin and Nestin under neurogenic inductive conditions for only 7 days *in vitro* (Figures 2(e) and 2(f)).

### 3.2. SHED Transplantation Decreased Cognitive Impairment in CCI Rats

**Figure 3:**
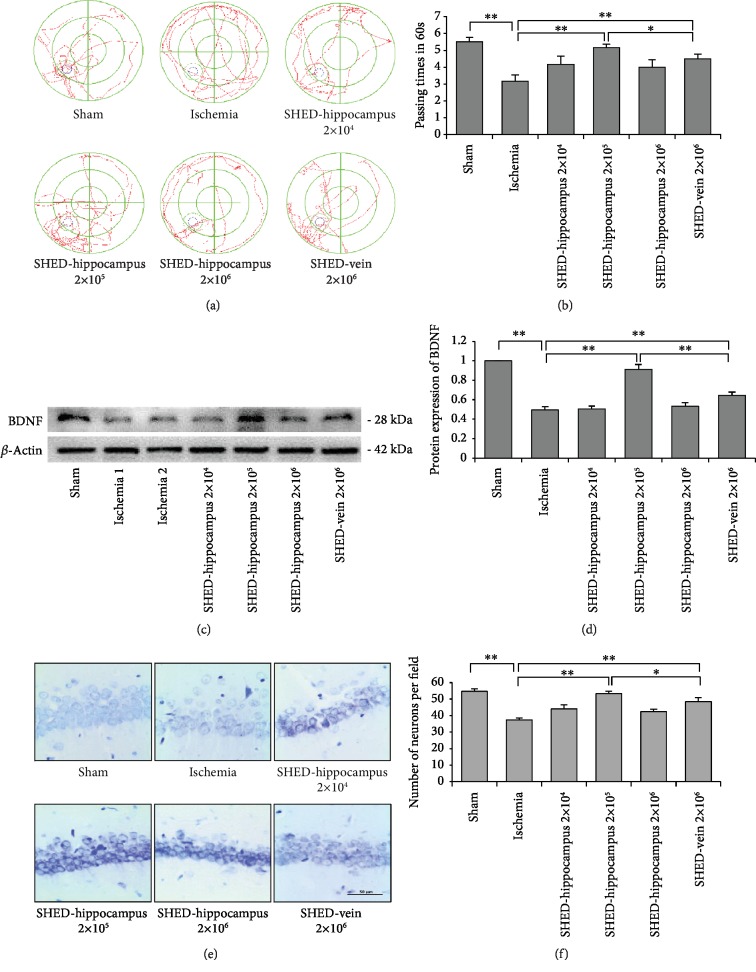
SHED transplantation improved the cognitive function and production of neurons in rats with chronic cerebral ischemia (CCI). (a, b) In the Morris water maze (MWM) test, SHED transplantation by hippocampal infusion (2 × 10^5^, ^∗∗^*P* < 0.01) or by tail vein injection (2 × 10^6^, ^∗∗^*P* < 0.01) increased the spatial memory of CCI rats compared with that in the ischemia group. (c, d) Western blot analysis showed that the expression level of BDNF was markedly increased in the SHED-hippocampus 2 × 10^5^ group (^∗∗^*P* < 0.01) and SHED-vein 2 × 10^6^ group (^∗∗^*P* < 0.01) compared with that in the ischemia group. (e, f) Nissl staining showed that SHED transplantation increased the number of surviving neurons in the hippocampal CA1 region compared with that in the ischemia group. Error bars: mean ± SD. ^∗^*P* < 0.05, ^∗∗^*P* < 0.01. Scale bar: 50 *μ*m.

CCI is characterized by progressive cognitive and behavioral deterioration. To evaluate the effects of SHED transplantation on cognitive deficits in CCI rats, we transplanted SHED inside the hippocampus or through the tail vein. The MWM test was used in this study. Compared to that in the sham group, spatial memory declined in the ischemia group. The number of passing times rats crossed the platform region in the ischemia group was significantly decreased in comparison to that in the sham group in the spatial probe test (*F*_(1, 10)_ = 37.69, *P* < 0.01); this effect was reversed by SHED transplantation either *via* the hippocampus or tail vein; there were significant differences between the ischemia and SHED-hippocampus 2 × 10^5^ (*F*_(1, 10)_ = 32.73, *P* < 0.01) or SHED-vein 2 × 10^6^ groups (*F*_(1, 10)_ = 12.31, *P* < 0.01); and there were differences between SHED-hippocampus 2 × 10^5^ and SHED-vein 2 × 10^6^ groups (*F*_(1, 10)_ = 5.71, *P* < 0.05). There were no significant differences between the sham and SHED-hippocampus 2 × 10^5^ (*F*_(1, 10)_ = 1.43, *P* > 0.05) (Figures 3(a) and 3(b)). These data indicated that SHED transplantation decreased cognitive impairment of CCI rats. Compared to the SHED-vein 2 × 10^6^ group, the effect of the SHED-hippocampus 2 × 10^5^ group was better on cognitive deficits in CCI rats. The detailed analysis is shown in Table 1.

The hippocampus has been considered a critical area in the brain that is related to cognitive functions, and hippocampal damages occurred in CCI rats after both carotid artery ligation [22]. Therefore, we further examined neuronal function in the hippocampus of rat brains after SHED transplantation. Western blotting analysis showed that the expression level of brain-derived neuronal factor (BDNF) was decreased in the ischemia group when compared with that in the sham group. SHED transplantation could increase the expression level of BDNF, the expression level of BDNF was increased the most in the SHED-hippocampus 2 × 10^5^ and SHED-vein 2 × 10^6^ groups compared with levels in the ischemia group (*F*_(1, 4)_ = 144.90, *P* < 0.01; *F*_(1, 4)_ = 28.96, *P* < 0.01) (Figures 3(c) and 3(d)), and there were significant differences between SHED-hippocampus 2 × 10^5^ and SHED-vein 2 × 10^6^ groups (*F*_(1, 4)_ = 57.56, *P* < 0.01). These data suggested that SHED transplantation promoted the recovery of neuronal function, the SHED-hippocampus 2 × 10^5^ group was better.

The number and function of neuronal cells in the hippocampus play an important role in spatial memory. Nissl staining was used to examine the neuronal number in the hippocampal CA1 region of CCI rats. Massively damaged neurons with pyknotic nuclei were observed in ischemic rats, and the number of surviving neurons reduced significantly compared with that in the sham group (*F*_(1, 4)_ = 245.82, *P* < 0.01). After SHED transplantation, the number of neuronal cells was increased in all SHED transplantation groups, including those in which transplantation was performed by hippocampal infusion and tail vein infusion. Furthermore, the number of neuronal cells increased the most in the SHED-hippocampus 2 × 10^5^ and SHED-vein 2 × 10^6^ groups compared to that in the ischemia group (*F*_(1, 4)_ = 209.46, *P* < 0.01; *F*_(1, 4)_ = 47.35, *P* < 0.01) (Figures 3(e) and 3(f)); and compared to the SHED-vein 2 × 10^6^ groups, the effect of the SHED-hippocampus 2 × 10^5^ group was better (*F*_(1, 4)_ = 8.65, *P* < 0.05). These data indicated that SHED transplantation markedly rescued the neuron number of the hippocampus in CCI rats.

### 3.3. SHED Transplantation Protected Hippocampal Neurons by Inhibiting Apoptosis

**Figure 4:**
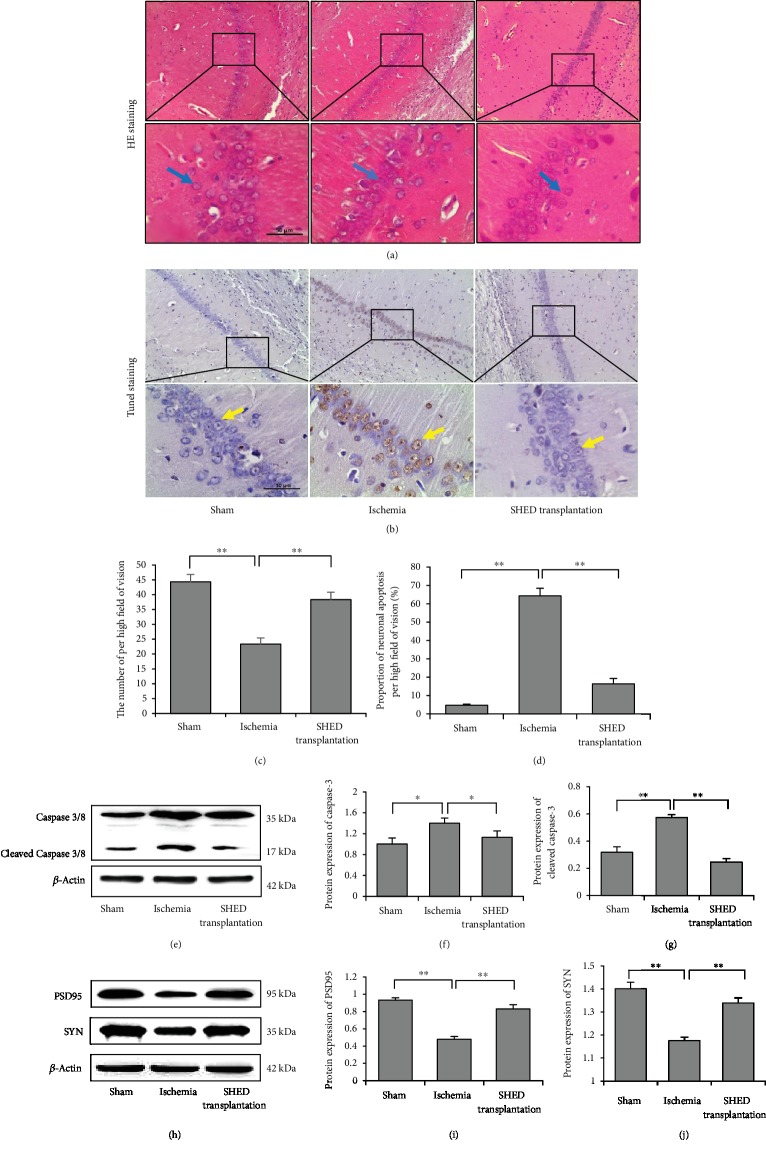
SHED transplantation enhanced neuronal function *via* inhibiting neuronal apoptosis in the hippocampal CA1 region of CCI rats. (a, c) HE staining showed that the number of neurons was decreased in the ischemia group, while SHED transplantation mitigated the neuronal loss (^∗∗^*P* < 0.01). Scale bar: 50 *μ*m. (b, d) TUNEL staining showed that TUNEL-positive neurons were present at significantly greater proportions in the ischemia group, while SHED transplantation decreased the proportion of apoptotic neurons (^∗∗^*P* < 0.01). Scale bar: 50 *μ*m. (e–g) Western blot analysis showed that the expression levels of caspase-3 and cleaved caspase-3 were markedly increased in the hippocampal tissue of the ischemia group, while SHED transplantation decreased the expression levels of caspase-3 and cleaved caspase-3. Error bars: the mean ± SEM. ^∗^*P* < 0.05, ^∗∗^*P* < 0.01 compared to the ischemia group. (h–j) Western blotting analysis showed that the expression level of postsynaptic density protein 95 (PSD95) and synaptophysin (SYN) was markedly decreased in the hippocampal tissues of the ischemia group, while SHED transplantation increased the expression levels of PSD95 and SYN. Error bars: mean ± SEM. ^∗∗^*P* < 0.01 compared to the ischemia group.

Since hippocampus transplantation of SHED had better therapeutic effects on CCI rats, so we used SHED transplantation *via* the hippocampus in the further experiments. HE staining showed that neuronal cells were arranged closely and were well organized with large and round blue-stained nuclei and that there were few spontaneous apoptotic cells in the sham group. In the ischemia group, the number of neurons was decreased, the distribution was uneven, the nuclear membrane was unclear, some of the nuclear membranes shrank, and coagulative necrosis and cell loss occurred. Compared to that in the ischemia group, SHED transplantation mitigated the neuronal loss in the hippocampal CA1 region of CCI rats (*F*_(1, 4)_ = 63.28, *P* < 0.05), and cell morphology and distribution tended to be normal (Figures 4(a) and 4(c)).

TUNEL staining showed that TUNEL-positive neurons, which were clearly observed as brown and sparsely scattered in the hippocampal section, were present at significantly greater proportions in the ischemia group (*F*_(1, 4)_ = 601.83, *P* < 0.01) (more than 60%). Additionally, the proportion of apoptotic neurons was dramatically decreased following SHED transplantation (*F*_(1, 4)_ = 259.20, *P* < 0.01) (Figures 4(b) and 4(d)).

Caspase 3/8, a member of the caspase family, can be activated by many factors and plays a vital role in apoptosis. In ischemia rats, the expression of cleaved caspase-3 and caspase-3 was increased, respectively (*F*_(1, 4)_ = 1069.89, *P* < 0.01; *F*_(1, 4)_ = 21.58, *P* < 0.05). SHED transplantation in CCI reduced the expression of cleaved caspase-3 and caspase-3 compared to that in the ischemia group, respectively (*F*_(1, 4)_ = 2447.46, *P* < 0.01; *F*_(1, 4)_ = 9.29, *P* < 0.05) (Figures 4(e)–4(g)). These results demonstrated that SHED transplantation protected hippocampal neurons by inhibiting their apoptosis.

Western blotting was performed to detect the protein expression of postsynaptic density protein 95 (PSD95) and synaptophysin (SYN), which somewhat indicated the neuronal function in the hippocampus of rat brains. In ischemia rats, the expression levels of PSD95 and SYN were decreased (*F*_(1, 4)_ = 477.95, *P* < 0.01; *F*_(1, 4)_ = 49.05, *P* < 0.01), and SHED transplantation increased their expression (*F*_(1, 4)_ = 125.67, *P* < 0.01; *F*_(1, 4)_ = 21.86, *P* < 0.01) (Figures 4(h)–4(j)). These results suggested that SHED transplantation promoted the recovery of neuronal function.

## 4. Discussion

In this study, we demonstrated that SHED transplantation protected neuronal cells and ameliorated cognitive functions in CCI rats when injected into the hippocampus or through the tail vein. Cui et al. reported that MSC transplantation could improve neuronal function [25]. The underlying mechanism of the therapeutic effects of SHED transplantation was mainly related to a reduction in neuronal apoptosis and a partial rescue of the cell function of damaged neurons. Spatial learning and memory capabilities are often used as an index to evaluate the cognitive function of rodent models. Spatial memory can be directly reflected by the observed passing times in a target quadrant within a certain time period. In this study, the MWM spatial probe test indicated that SHED transplantation partially reversed cognitive impairment in rats after the induction of CCI. Previous studies have reported that bone marrow mesenchymal stem cells (BMMSCs) can play a neuroprotective role and improve the learning and memory ability of CCI rats [9]. However, SHED show a stronger proliferation than BMMSCs and have a multidirectional differentiation potential, in particular, a strong neuronal differentiation ability, as well as significant immunomodulatory effects [26, 27]. SHED, as unique mesenchymal stem cells, have a low immunogenicity and can be used for allogeneic or heterogeneic transplantation [28]. Moreover, SHED can be obtained from a wide range of sources through noninvasive methods. Therefore, SHED show great potential for clinical applications as an excellent source of stem cells.

The long-term cerebral blood perfusion insufficiency in CCI rats results in a lack of oxygen and glucose and inflammatory response in the brain, causing cell apoptosis of neurons [29, 30]. In previous researches, it was found that SHED transplantation regulated the balance between the proapoptotic factor tumor necrosis factor-*α* (TNF-*α*) and the antiapoptotic factor Bcl-xl, reduced early neuronal apoptosis, and caused a recovery of spontaneous motor function as early as 1 week after spinal cord injuries in rats [31, 32]. Another report showed that SHED transplantation reduced neuronal apoptosis, inhibited the expression of the proinflammatory cytokines TNF-*α* and interleukin- (IL-) 1*β*, increased the expression of the anti-inflammatory cytokines IL-4 and IL-10, and improved the survival of perinatal hypoxia-ischemia mice [33]. Traumatic brain injury (TBI) has similar mechanisms to CCI, and it has been reported that SHED rescued motor function and reduced neuroinflammation in TBI rats, with the therapeutic effects being related to exosomes derived from SHED [20]. In addition, it has been suggested that SHED or SHED-derived conditioned medium could play a neuroprotective role in neurons and improve neuronal function in Parkinson's disease and AD [19, 34]. These paracrine effects provide a new insight into the therapeutic effects of SHED transplantation. The present study further confirmed the neuroprotective effect of SHED against CCI, another major type of neurological disease.

Currently, the pathogenesis of CCI is unclear. It may be related to neuronal damage, synaptic abnormalities, neurotransmitter dysfunction, energy metabolism disorders, or other factors. Moreover, neurological diseases are mostly caused by decreases in neuron production and functional neuron deficits. We found that SHED transplantation could reduce neuronal apoptosis and decrease neuronal injury in rats with CCI. BDNF, a member of the neurotrophin family of nerve growth factors, is actively produced throughout the brain and is involved in neuronal development, differentiation, and survival. BDNF plays a central role in modulating synaptic plasticity in the developmental process of the brain [35]. BDNF has been highlighted as a key regulator of functional recovery and was reported to have significant protective effects against ischemic brain disease [36–38]. The spatial reference memory of mice with partial BDNF knockdown was shown to be impaired [39], and it has been reported that functional recovery after an ischemic stroke was most often associated with increased BDNF expression and that reduced BDNF expression resulted in diminished neural plasticity and functional recovery [40–43]. In the early stages after ischemic injury, BDNF is elevated in tissues surrounding the injured site following the major loss of neurons [43, 44]. However, the relative levels of BDNF after ischemic injury may not be sufficient to overcome barriers and aid in functional recovery. MacLellan et al. identified the critical threshold of BDNF expression needed to facilitate enhanced neuronal plasticity and post-stroke recovery [45]. A recent research reported that transplanting BMMSCs through the tail vein increased BDNF expression in the infarcted hemisphere of the brain and elicited functional recovery in rat stroke models [46]. In the present study, we found that SHED transplantation significantly increased BDNF levels in the brain of CCI rats. Therefore, improving neuronal plasticity of rescued neurons might contribute to the decrease of cognitive impairment from CCI after SHED transplantation.

Presynaptic and postsynaptic proteins are activated by BDNF and play a vital role in synaptic plasticity in the hippocampus [39]. Synaptic plasticity-associated proteins, including SYN and PSD95, have been shown to be involved in many neurological diseases owing to their important role in hippocampal structural plasticity. It is well known that cognitive deficits in AD are caused by synaptic dysfunction. Research has shown that *L*-3-n-butylphthalide, an extract from the seeds of *Apium graveolens* (Chinese celery), increased the expression of PSD95 and SYN, attenuated the development of A*β* plaques and neuroinflammatory responses, promoted hippocampal neurogenesis, and improved behavioral recovery in aged AD mice [47]. Another study showed that the expression of SYN and PSD95 was upregulated in SMAP8 mice after 14 days of stimulation by repetitive transcranial magnetic stimulation; this alteration in synaptic biomarkers was accompanied by improved cognitive function in these mice [48]. In the present study, it was found that the expression of PSD95 and SYN was decreased in the hippocampus of CCI rats and that SHED transplantation promoted neurological recovery by upregulating the expression of BNDF, PSD95, and SYN.

Previous studies have suggested that MSC transplantation had neuroprotective effects against neurological diseases. However, the number of transplanted MSCs that reaches the hippocampus/paracele has not been determined and ranges from 5 × 10^3^ to 3 × 10^6^ cells [36, 49, 50]. In this study, a gradient dose was administered for hippocampus transplantation, and we found that the appropriate dose of SHED transplantation for alleviating CCI in rats was 2 × 10^5^ cells/10 *μ*L. Since the volume for hippocampal infusion is strictly limited, a high dose of SHED may result in insufficient resuspension and cell clumps, causing immune responses locally and decreasing the therapeutic effects of SHED transplantation. Our study provided a theoretical basis for further research regarding their applications. In this study, we also observed a small quantity of green positive cells near blood vessels in the lesioned hippocampal area after PKH67 labelled SHED transplantation *via* the tail vein, which might be due to the destruction of the blood-brain barrier by CCI (data not shown).

## 5. Conclusions

SHED transplantation successfully promoted neurological function by inhibiting neuronal apoptosis and upregulating the expression of neurologically relevant proteins. As significant stem cells with great potential, SHED showed a promising clinical value for the treatment of neurological diseases.

## Figures and Tables

**Table 1:** SHED transplantation decreased cognitive impairment of CCI rats (MWM).

Group	Passing times in 60s						Average
Sham	5	5	6	6	6	5	5.50±0.55^∗∗^
Ischemia	3	3	4	2	4	3	3.17 ± 0.75
SHED-hippocampus 2 × 10^4^	4	4	3	4	4	6	4.17 ± 0.98
SHED-hippocampus 2 × 10^5^	5	5	5	5	5	6	5.17±0.41^∗∗^
SHED-hippocampus 2 × 10^6^	5	5	4	3	4	3	4.00 ± 0.89
SHED-vein 2 × 10^6^	5	5	4	4	5	4	4.50±0.55^∗∗^

Error bars: mean ± SD. ^∗∗^*P* < 0.01 compared to the ischemia group.

## Data Availability

The data used to support the findings of this study are available from the corresponding author upon request.

## Supplementary Materials

Supplementary Materials Supplementary Figure 1: the change in blood flow velocity after both common carotid artery ligation.
