# Supplementary material for: Transplantation of Stem Cells from Human Exfoliated Deciduous Teeth Decreases Cognitive Impairment from Chronic Cerebral Ischemia by Reducing Neuronal Apoptosis in Rats
Source: Stem Cells Int. 2020 Mar 6;2020:6393075. doi: 10.1155/2020/6393075 (PMC7079222; doi:10.1155/2020/6393075)
Supplement: Supplementary Materials — Supplementary Figure 1: the change in blood flow velocity after both common carotid artery ligation. [file 6393075.f1.pdf]

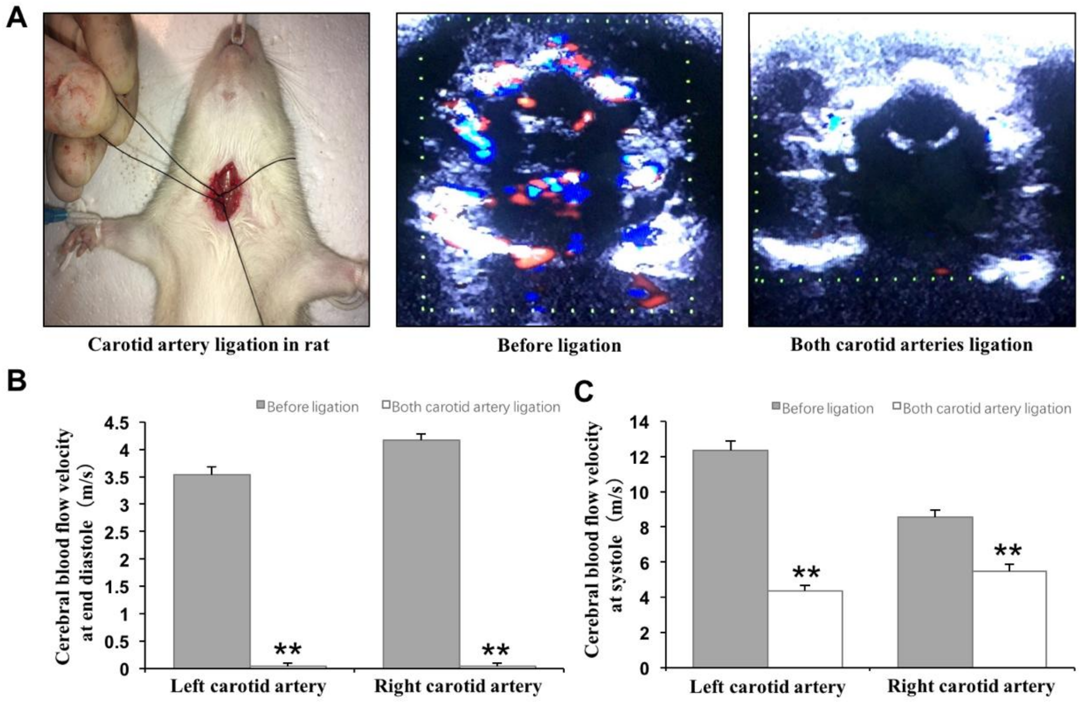

Supplementary Fig. 1. The change in blood flow velocity after both common carotid artery ligation. (A) The ultrasound images after both common carotid artery ligation. (B) Changes in cerebral blood flow velocity of both common carotid arteries at end diastole. (C) Changes in cerebral blood flow velocity of both common carotid arteries at systole. Error bars: mean ± SEM. $^{**}P < 0.01$.
